# Dignity of Older Persons With Mental Health Conditions: Why Should Clinicians Care?

**DOI:** 10.3389/fpsyt.2021.774533

**Published:** 2021-11-12

**Authors:** Debanjan Banerjee, Kiran Rabheru, Gabriel Ivbijaro, Carlos Augusto de Mendonca Lima

**Affiliations:** ^1^Psychiatry, Private Practitioner, Kolkata, India; ^2^University of Ottawa, International Longevity Centre (ILC), Ottawa, ON, Canada; ^3^Department of Psychiatry, NOVA University, Lisbon, Portugal; ^4^Department of Psychiatry, The Wood Street Medical Centre, London, United Kingdom; ^5^Old Age Psychiatry Section, World Psychiatric Association, Geneva, Switzerland

**Keywords:** dignity, human rights, older people, ageism, elder abuse, mental health

## Abstract

With a steady increase in population aging, the proportion of older people living with mental illness is on rise. This has a significant impact on their autonomy, rights, quality of life and functionality. The biomedical approach to mental healthcare has undergone a paradigm shift over the recent years to become more inclusive and rights-based. Dignity comprises of independence, social inclusion, justice, equality, respect and recognition of one's identity. It has both subjective and objective components and influences life-satisfaction, treatment response as well as compliance. The multi-dimensional framework of dignity forms the central anchor to person-centered mental healthcare for older adults. Mental health professionals are uniquely positioned to incorporate the strategies to promote dignity in their clinical care and research as well as advocate for related social/health policies based on a human rights approach. However, notwithstanding the growing body of research on the neurobiology of aging and old age mental health disorders, dignity-based mental healthcare is considered to be an abstract and hypothetical identity, often neglected in clinical practice. In this paper, we highlight the various components of dignity in older people, the impact of ageism and mental health interventions based on dignity, rights, respect, and equality (including dignity therapy). It hopes to serve as a framework for clinicians to incorporate dignity as a principle in mental health service delivery and research related to older people.

## Premise: Dignity In Mental Health Care For Older People

The world's population is aging rapidly with persons aged 65 or older projected to reach 1.5 billion by 2050 (1). Approximately 20% of them will have mental health conditions such as dementia, depression, anxiety and substance use, often complicated by physical and psychosocial comorbidities (2). Implicit and explicit biases that negatively influence their care include the triple jeopardy of ageism, mentalism, and ableism (3). The concept of dignity is complex and forms the ethical basis for enhancing a person's sense of wellbeing and quality of life, especially for persons with mental health conditions. Our neurobiological understanding of late-life mental health conditions has improved significantly over the last few decades, but there is an urgent and significant unmet need to incorporate the principles of dignity within mental health service delivery (4). This is particularly important to address for clinicians caring for older persons who must respect the human rights and the autonomy of every person. Incorporating dignity in the care of older persons takes on greater importance, due to their multiple and interdependent vulnerabilities such as physical, psychological, cognitive, and social frailty, interacts with dependence on others, loneliness, social isolation, polypharmacy, medical comorbidities subjecting them to human rights abuses, loss of autonomy and poorer access to healthcare. Promoting their dignity and protecting older persons against stigma, discrimination, violence, abuse and neglect enhances clinical outcomes and quality of life.

Practical models for promoting the principles of dignity, and a rights-based approach to mental health care, serve as a moral, ethical, and legal anchor to support the independence and autonomy of older persons with mental health conditions (5–7). Most older persons have higher ratings of successful aging despite declining physical and cognitive function and satisfied with their quality of life (8), however with speedy population aging, there are several others with increased needs of support. These include people with chronic medical illnesses, geriatric depression and anxiety, as well as neurocognitive disorders. Besides functional recovery, optimum management of their conditions also needs to preserve their independence, respect, autonomy and rights. Even though most of these concepts are of a Western origin, transcultural connotations are common. This assumes a special significance in light of the United Nations Convention for Rights of People with Disabilities (UNCRPD), which views human rights as an “*instrument with an explicit, social development dimension”* (9). There are currently 82 signatories to the Convention who agree that fundamental freedoms should be at the core of healthcare in conditions of disability, which include psychiatric disorders. The UNCRPD changes the approach from viewing the “*mentally disabled”* as “subjects of charity needing medical and social protection” to “individuals with human rights who can participate in society and informed decision-making with appropriate care” (10). This furthers the concept the autonomy and free will beyond the geographical and cultural boundaries. Besides, autonomy in a mental health setting, but also in other contexts of vulnerability, is often an ideal that can become a fallacy if structural factors are ignored; in some settings, the ideal of autonomy should be framed as interdependency (11–13). Controversies about the cultural acceptability of the concept of “autonomy” aside, it is only one of the dimensions of dignity, which is a much more holistic concept in healthcare. The social context of care, process of caregiving, challenges associated with aging and psychiatric symptoms: all can be potentially grounded in recognition of the individual's abilities, respecting the free will, preventing health inequalities and recognition of diversities, all of which constitute dignified mental healthcare in daily practice (14). As global health inequalities are widening, more so in light of the ongoing Coronavirus Disease 2019 (COVID-19) pandemic, incorporating dignity in mental health services and planning become more important. Non-inclusion of such practices in mental health policies and programmes have led to poor healthcare access, stigma, inadequate social welfare benefits and discrimination in older people (15, 16).

## What Constitutes Dignity: Different Dimensions

Dignity is a complex multi-dimensional construct with a high likelihood for various subjective interpretations. It is difficult to have an universally agreed definition for such an abstract concept. Nevertheless, people are usually able to recognize when an individual's dignity is violated, and also when dignity in care is enhanced. Hence, when we talk about the need for dignity in mental health, there needs to be a shared understanding among clinicians, patients, caregivers and policy-makers alike (14, 17).

Dignity has often been conceptualized in sync of human rights in being of “*value or worthy”* and enjoying the “*deserved respect.”* The United Nations Universal Declaration of Human Rights states that “*All human beings are born free and equal in dignity and rights. They are endowed with reason and conscience and should act toward one another in a spirit of brotherhood”* (18). This is translated in the Constitution of the World Health Organization (WHO) when it is stated that “*The enjoyment of the highest attainable standard of health is one of the fundamental rights of every human being without distinction of age, race, religion, political belief, economic or social condition”* (19).

In the practice of medicine, dignity gradually became a focus for physicians and medical researchers. In more recent debates, it has been invoked in questions of bioethics of human genetic engineering, human cloning, and end-of-life care (20). In June 1964, the World Medical Association issued the Declaration of Helsinki that says at article 11: “*It is the duty of physicians who participate in medical research to protect the life, health, dignity, integrity, right to self-determination, privacy, and confidentiality of personal information of research subjects”* (21). The Council of Europe, on 4th April 1997, at Oviedo, approved the Convention for the Protection of Human Rights and Dignity of the Human Being with regard to the Application of Biology and Medicine. The Convention states that “*Parties to this Convention shall protect the dignity and identity of all human beings and guarantee everyone, without discrimination, respect for their integrity and other rights and fundamental freedoms with regard to the application of biology and medicine”* (22).

A phenomenological analysis of how care providers perceived dignity as a core value in healthcare promotion in older people revealed that it was constituted by three primary components: worthiness, autonomy, and identity (23). The authors state that these themes reflected the principles of nursing practice to improve the older patient's health potential. Ivbijaro et al. (24) highlighted that dignity is not simply a medical strategy but rather a holistic outlook to healthcare which involves both service users and providers. They mention cultural sensitivity, kindness, respect, empathy, independence, autonomy and self-esteem as the various dimensions of dignified mental healthcare for older people. Targeting these attributes in various settings through collaborative care can enhance the quality of life of seniors who are mentally ill. Further, principles of dignity can also help counteract ageist attitudes, negative beliefs and social stereotypes about “being old” both among the general public and mental healthcare professionals (25). It's also vital how service consumers understand dignity themselves. A recent meta-synthesis explored the “understandings of dignity” from the perspective of older adults in the Nordic countries (26). The need for recognition and visibility formed the overarching theme and also an important unmet need in healthcare. There was a critical need to balance between “*toning down their illness to gain more independence”* vs. “*losing a voice by being less visible to the society.”* Hence, besides autonomy and independence, the need for social visibility also formed a vital component of dignity. In lines with the same, mental health first aid delivery based on dignity has shown to be an effective public health intervention tool for improving knowledge, attitude and practices (27). Informed decision-making, participation in healthcare, providing audience to the voices of persons with mental illness and a non-judgemental approach formed the main components.

Based on this evidence, personal dignity can be understood in two interconnected ways: *internal* (how I see myself) and *external* (how others see me) (28). Each is dependent on the other. Hence, as a mental health professional it is essential to have an inclusive, empathetic and non-discriminative approach to consider the individual as “*an independent human being”* rather than just a “*subject of care.”* This brings in the sense of “subjectiveness” and “individualism” which are important constructs of self-dignity (26). The temptation to stereotype older adults into a single homogenous group takes away their individuality, contributes to the increased stigma associated with older age and increases the risk of denying them their dignity. Based on ethical principles, dignity has also been conceptualized as autonomy/self-determination, non-maleficence, ensuring justice, and veracity (right to know and participate in their treatment process) (20).

It is clear from the discussion above that dignity involves various attributes which reinforce each other. Also the perceptions vary based on needs and care-providing. Unfortunately, most of the interventions and guidelines speak about “*needs and should”* in dignified mental healthcare rather than highlighting the processes of change (28, 29). Dignity in true sense is both structural and interpersonal. These dynamics need to be considered while imbibing it into mental health interventions. A common fallacy is attributing lack of dignity solely to psychological causes while it is divorced from “symptoms” which essentially have a medical model. This dichotomy is potentially harmful (30). It is high time that the premise of dignity and rights in mental healthcare of older adults are considered through a biopsychosocial model. Maintaining respect, optimum medication to ensure functional independence and granting social recognition as an individual go hand-in-hand and hence no single dimension of personal dignity can exist in a vacuum. As Clancy et al. (26) state that the effect of mental illness is to make older people invisible to the society and their voices unheard, it is the onus of healthcare providers to protect them from this “*cloak of invisibility.”*

## Dignity: How Does It Intersect With Psychosocial Health

**Figure 1:**
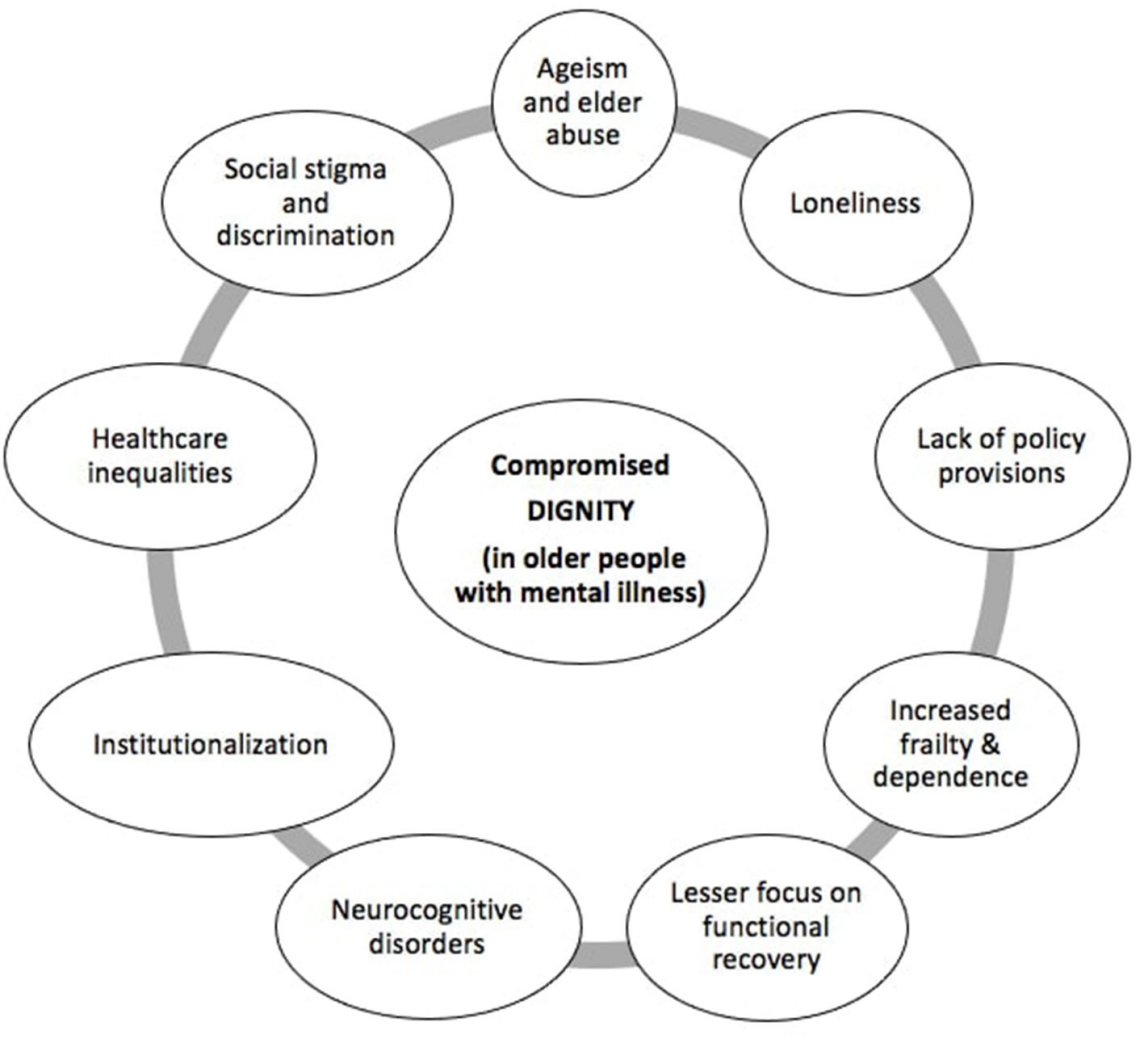
Various factors that impose threat to dignity in older people living with mental illness.

Collaborative care is the cornerstone of mental health services in older people. This requires both empathy and community support. There is growing evidence that clinical empathy–the medical professional's cognitive understanding of the emotions of people with mental illness combined with emotional attachment–directly enhances therapeutic efficacy (31). Increasingly, training in empathetic behavior must be a priority among healthcare professionals caring for the elderly (32). While delivering mental health services for older people, it is important to consider the social determinants of health, which are dynamic throughout the life-course, especially in later life. Social determinants of health are the conditions in which people are born, grow, live, work and age and which are shaped by the distribution of money, power and resources at global, national and local levels (33). These undergo complex interaction with genetic factors, personal experiences as well as the social environment to influence psychological wellbeing. The various dimensions involved in mental illness that also influences personal dignity and human rights in older people are depicted in Figure 1.

Social determinants may play a role as risk factors for mental health problems (unemployment, poverty, inequalities, stigma and discrimination, poor housing, adverse childhood experiences, violence, abuse, drug and alcohol abuse, poor general health, caring duties), while others may be protective factors (social protection, resilience, social networks, positive community engagement, positive spiritual life, hope, optimism, good general health, good quality family interactions, positive intergenerational relationships) (34, 35).

By acting on social determinants of health, it is possible to contribute to promote the older adults' dignity and a better subjective mental health and well-being of older people, to build the capacity of communities to manage adversity, and to reduce the burden and consequences of mental health problems. Disadvantages because of mental health problems in old age damage the social cohesion of communities and societies by decreasing interpersonal trust, social participation and civic engagement (36). The present-day nosology-focused symptom-triggered psychiatric care often aim at relieving the signs of illness and reduce hospitalization. This can at times deprive the voices of the service-users with their exclusion from the management plan. According to Minoletti (37)

“*dignity for persons with mental disorders is exercising citizenship, with a sense of empowerment and control over their lives, and demanding the same rights (e.g., the right to decide where to live, whom to meet, whom to love, where to work, etc.) and take the same responsibilities (e.g., respecting the laws, voting, volunteering, paying taxes, etc.) as other citizens.”*

Hence, besides symptom resolution, enjoying citizenship, feeling respected (as well as self-respected), honored, and inclusive are vital components of care in a mentally ill older adult. This makes the resultant mental healthcare for older adults comprehensive and patient-centered rather than bureaucratic and directive.

## Ageism And Elder Abuse: Fundamental Threats To Human Rights And Dignity

Ageism manifests in various forms throughout the life course and is most impactful in old age. It is the stereotyping, prejudice and discrimination against an individual based on his/her age or the aging process (38). Coined in 1969 by Robert Butler, it primarily denotes ageist attitudes against senior citizens, although it is also rooted in sexism and racism (38). The World Health Organization (WHO) Global Report on Ageism was developed by the High Commissioner for Human Rights, the United Nations (UN) Department of Economic and Social Affairs as well as the United Nations Population Fund (UNPF) (39). The report calls upon healthcare policies, intergenerational and multi-disciplinary interventions to combat ageist behavior against older people, considering ageism as a major barrier to personal dignity and social visibility (39). Ageism is widespread across places providing health and social care, in workplace, the media, etc. to an extent nearly 50% of the global population have some or the other variation of ageist thoughts, which is much more prevalent in the low-and-middle-income countries (40). Ageism has shown to compound the stigma against mental illness, especially in neurodegenerative disorders as old age is often equated with the process of “*disease and decay.”* This can lead to a “*double jeopardy”* in the psychiatrically ill older population (41). Human-rights based approach targeting inequalities and discrimination in healthcare can restore inclusion, dignity and autonomy in psychogeriatric care and thus address the long-term issue of ageism.

Ageism also leads the concerning social evil of elder abuse, which is high in institutional settings. Based on WHO data, one in six individuals above 60 years age have experienced some form of abuse in the community settings within the last year (42). Ageism affects not only the older adults mentally ill but also who care them. Often it is perceived that care older adults with mental health conditions less prestigious than other positions in the health care systems, with prejudice for the professionals' career, self-esteem and their own mental health (42). Family members of older adults with mental health condition may also be victim of stigma and discrimination, with loss of personal projects and resources (including financial ones). Both ageist attitudes and consequent elder abuse have peaked during the COVID-19 pandemic and their intersections with human rights crisis, marginalization and loss of dignity in older people are quite prominent (43–45). Banerjee et al. (46) propose the complex interplay of ageism (and other forms of discrimination in older people), self-stigma, health inequality, and elder abuse that leads to compromised dignity which in association with factors such as frailty, dependence, social isolation and medical morbidities lead to “human rights crisis” in older people. The processes of “*rudeness, dismissal, indifference, disregard, objectification, condescension, intrusion, restriction, labeling, discrimination, contempt, deprivation, abjection, revulsion, and assault”* lead to violation of dignity in healthcare and reinforce ageist behaviors in the service providers (47). Incorporation of principles such as care, empathy, respect for individual identities, focus on safety, non-judgmental approach and social justice into geriatric mental healthcare can help combat ageism and elder abuse in lines with the Global Strategy and action plan on aging and health and the Decade of Healthy Aging 2021–2030 (48, 49).

## What Is Warranted In Mental Healthcare And Areas Of Intervention?

Most older people want the same as everybody else in the general population. They want services that are reliable and dependable, easy to access and with competent staff that are sensitive and recognize diversity. When they have health needs, older adult people like to have these needs managed in a collaborative way (50). This requires a supportive community. Older people want to be close to their families if they need to receive treatment in hospital (8, 24, 35). We need innovation for this to be achieved, taking the patient's view into account and may have to adapt and use new technology in an ethical and responsible way. A review of empirical and theoretical literature on dignity in the care of older people revealed that staff attitudes and behavior, environment, culture of care, specific care activities and staff training form the major factors in deciding personal dignity. Sense of purpose and need for recognition in daily life were important considerations for dignity while in mental healthcare there was a constant tussle between degree of dignity and autonomy especially in severe mental illness and dementia (37, 43, 46). The practical wisdom of mental health professionals, social workers, case managers and policy makers and their perceptions about what constitutes dignity are vital in determining the operational interventions at place in mental healthcare settings for older people (37, 46).

Health and social professionals need to receive special education and training to care older adults with mental disorders with dignity. To educate professionals, caregivers and the lay public in mental health issues in old age is necessary to reduce the burden of mental disorders. All health and social professionals should receive information according to a knowledge based curriculum on mental health issues in old age at undergraduate and postgraduate levels. Such curriculum should include the special significance in old age of the interdependence of mental, physical and social factors and the prevention and health promotion including recreational and spiritual issues (51, 52). Recovery-oriented and person-centered practices need to be at the core of healthcare. The various areas through which principles of dignity can be incorporated in psychosocial care for older people are mentioned in Table 1.

**Table 1:** General areas of dignity-based mental health interventions.

- Addressing stigma and discrimination in individuals with mental illness - Combat ageism - Prevention and management of elder abuse - Day care facilities and housing security - Vulnerable populations (low socio-economic status, homeless, sexual minorities, migrants and displaced populations, nursing home residents [Table 2], etc.) - Management of physical/social frailty and falls - Ensure privacy and sexual health - Optimizing psychiatric medication and focus on functional recovery/independence - End-of-life considerations and informed decision making - Dignified care in neurocognitive disorders - Use of technology (digital literacy) - Research in dignity-based mental healthcare/lived experiences of consumers and their caregivers - Focus on respectful communication and social recognition as “individuals” (rather than patients) - Inclusion of older people with mental illness/their families in interventions and policies - Work with other sectors of society (justice, welfare, security forces, economy, etc.) to promote protection against ageism and to prevent ageist attitudes.

### Sheltered Housing, Residential, and Nursing Care Homes and Hospitals

Many older people will be requiring nursing, residential and sheltered homes. Research shows that well-trained staff sensitive to their dignity and independence provide them emotional security, better quality of life and reduce risk of falls (50). Religious, spiritual and cultural values also need to be considered and incorporated into care planning (7, 53, 54). Evidence-based facets of dignity-promoting interventions in nursing home/residential settings are summarized in Table 2.

**Table 2:** Ensuring dignity-based care for older people in nursing homes/residential facilities (50, 55–59).

•Optimal pain management • Improving communication within and outside the facility (in-person and digital) • Respect in daily conversation • Foster independence in functioning (self-care: choice of living, eating, dressing, etc.) • Good nutrition and hydration • Ensure decent physical appearance, personal and oral hygiene • Healthy interactions with the staff • Age-friendly environment for mobility and safety • Supervision for security and prevention of abuse • Adequate ward design for ensuring privacy and sexual rights • Group activities, exercise and engagement within the facility • Prevent loneliness and isolation • Restrict empirical use of psychotropic medications • Residential care plan to include principles of dignity • Involvement in decision-making to the extent possible • Special care for those with severe mental disorders and dementia • End-of-life care (avoid unnecessary and painful prolongation of life) • Dignity therapy for older persons and their families in case of comorbid terminal illness

Independence is to be encouraged. More sensibility is needed in order to understand needs and desires of people for whom it is difficult to verbalize their preferences, such as in advanced dementia, confusional states and others. Such approaches generally need well-trained staff who knows well the older individual and his or her life history (50).

Hospital managers need to play a role in ensuring that the institution promotes dignity and that staff have the capabilities to provide dignity in care, for instance by avoiding treating older people like children. Ward design can either support privacy, autonomy and dignity or make patients more vulnerable by making it harder to promote individuality (54, 60, 61). Research has suggested that bringing in pieces of their own furniture and household belongings including photographs supports the recognition of individuality (50, 53, 62).

One of the greatest challenges to care delivery in sheltered, residential and nursing care settings is that of balancing risk management with privacy whilst supporting maximum independence. There need to be environmental, relational, and procedural structures in place to ensure that the older adult have as much independence and privacy as possible whilst reducing risk because many people's greatest fear when they enter such accommodation is loss of independence (54, 60).

While good staff communication skills are fundamental to the promotion of dignity in older adults, this is a skill that can be trained. Older people living with mental illness should routinely be asked about how they would like to be addressed, and there should be meaningful interactions between those who care for the older adult and the older adult being cared for in order to avoid social isolation (59).

### Dementia

In addition to the treatment interventions offered, an important goal in the care of dementia is supporting quality of life, dignity and comfort: this should remain central to treatment and care delivery. Meaningful attention should be paid to the activities of daily living, the choice of treatments offered and the involvement and engagement of the individual and their family to enhance and maintain the individual's dignity (63).

Intervention programme that include the individual's family network is helpful. Family care giver's health needs should always be considered: positive health in family care givers may improve the well-being of the person with dementia and prevent care givers burn out affecting their own mental health balance. This promotes dignity by offering opportunities to care persons at the place of their choice (64).

### Decision-Making and Dignity

It is necessary to develop national frameworks to protect people with impaired decision-making ability, in the respect of the person's dignity, and in accordance with the article 12 of the UN Convention on the rights of persons with disabilities (10). Substitute decision making (SDM) arrangements including informal surrogate through proxies appointed by the care recipient when still competent to those who are Court-appointed. SDM measures and actions must be in the interests of the incapacitated person and their continuing necessity should be reviewed regularly. They can be tailored based on national policies and socio-cultural norms. Mechanisms should be in place for appeal and for review as well as for reporting of alleged mistreatment by SDMs (20).

### Recommendations: Role of Dignity Therapy

The population is aging and multimorbidity and co-morbidity is no longer the exception. People who care for older adults must be willing to understand the effects of multimorbidity and co-morbidity to promote dignity and the rights of older adults.

The quality of care people with dementia receive in hospital has raised concerns in many quarters and the need for clinicians to focus on dignity for people in hospital or receiving end of life care is very important (65). In addition to having a legal framework to support dignity and the rights of older adults we also need to provide practical tools for those who care for older adults to use. Applying these tools will help the older adult to maintain their autonomy and improve their self-identity and sense of purpose.

Dying with dignity in older people is very important to relatives and to the individual concerned and the end of life presents a further challenge to older adults (66). Factors that are associated with dignity in death for an older person are complex and include the type of multi and co-morbidity, quality of relationships with family including siblings and with caregivers, enjoyment of good days prior to death, feeling contented, not feeling lonely or a burden and maintaining a feeling of being in control. We need to ensure that end of life care promotes a sense of dignity and purpose and Dignity Therapy (DT) as a psychotherapeutic intervention for people near the end of life has been explored and has the potential to enhance the dignity of the older person.

A 2005 Canadian study showed that 91% of participants were satisfied with DT with 75% of participants reporting an enhanced sense of dignity (67). DT is one of the few non-pharmacological interventions that can be useful in older adults at the end of life however this is not routinely taught in clinical practice and we should recommend this approach as an additional tool in the armory of clinicians working with older adults in end of life care.

DT is not new and is well-accepted by family members (68). Sixty family members of people who had participated in DT and later died were surveyed to understand their perspectives on DT and 95% of respondents said that it helped their loved ones and 78% reported that it had heightened their loved one's sense of dignity and reduced their suffering (68). 65% of the family members felt that this form of therapy constitutes the most important part of healthcare and nearly all of them wanted to recommend it to other families confronting a terminal illness.

A randomized controlled trial in patients with a terminal prognosis receiving palliative care in hospital or community setting compared DT with client-centered care and standard palliative care (69). Though reduction of patient distress was same in all the three interventions, DT was significantly more likely to improve family's stress, quality of life and sense of purpose in those affected. A 2015 systematic review of DT concluded that there is robust evidence to support its acceptability and suggested the need for further research into how and in what settings it should be provided (70). Another systematic review of DT in palliative care by Martínez et al. (71) showed that DT was effective in reducing psychological distress, enhancing resilience and improving depression and anxiety scores. Non-randomized studies have reported significant gains in existential distress, death anxiety and psychosocial measures, which warrant further study. Even though, DT has been used mostly in palliative care, the principals involved be translated to routine clinical care of older people so that inclusion, compassion, sense of purpose, quality of life and person-centered approach can be used rather than a purely symptom-triggered diagnosis-based management.

## Way Forward: Call For A Un Convention On Rights For Older People

As professionals from different backgrounds and regions of the world we collectively pledge to employ a human rights-based lens aimed to reduce the burden of ageism, mentalism, and ableism permeating virtually every aspect of older persons' lives (3). “Leave no one behind (LNOB)” is at the heart of the United Nations' (UN's) 2030 Sustainable Development Goals (SDGs) (72). The UN's Decade of Healthy Aging (2021–2030) strives for all persons to enjoy peace, prosperity, and a healthy planet (48). These commendable aspirations are clearly not the reality of countless older persons who have suffered needlessly for decades. “*What you permit, you promote.”* Let us not permit this status quo. Let the lives sacrificed by older persons not be in vain by ensuring a positive change for the human rights of every generation of older persons. A global paradigm shift is required to transform the deeply-rooted stigma against older persons to one where every older person can fully enjoy their life with dignity and with respect. An urgent call for action is needed for a legally binding United Nations convention on the human rights of older persons.

## Data Availability Statement

The original contributions presented in the study are included in the article/supplementary material, further inquiries can be directed to the corresponding author.

## Author Contributions

DB drafted the first version of the manuscript with contributions from KR, CM, and GI who also edited the manuscript. The final version was read and approved by all the authors. All authors were involved in conceptualization.

## Conflict of Interest

The authors declare that the research was conducted in the absence of any commercial or financial relationships that could be construed as a potential conflict of interest.

## Publisher's Note

All claims expressed in this article are solely those of the authors and do not necessarily represent those of their affiliated organizations, or those of the publisher, the editors and the reviewers. Any product that may be evaluated in this article, or claim that may be made by its manufacturer, is not guaranteed or endorsed by the publisher.
